# Experimental investigations into the performance of die-sinking mixed-gas atomization discharge ablation process on titanium alloy

**DOI:** 10.1038/s41598-022-06457-4

**Published:** 2022-02-14

**Authors:** Linglei Kong, Weining Lei, Qun Wei, Jinjin Han, Zhang Suorong, Qilin Li, Xiangzhi Wang, Zhidong Liu

**Affiliations:** 1 School of Mechanical Engineering, Jiangsu University of Technology, Changzhou, 213001 China; 2 College of Mechanical and Electrical Engineering, Nanjing University of Aeronautics and Astronautics, Nanjing, 210016 China; 3 Jiangsu Key Laboratory of Non-Traditional Machining, Changzhou Institute of Technology, Changzhou, 210032 China

**Keywords:** Aerospace engineering, Mechanical engineering

## Abstract

As a variant of highly efficient electrical discharge machining (EDM), the die-sinking mixed-gas atomization discharge ablation process (DMA-DAP) uses an atomized dielectric formed by a mixed gas, which mainly composed of oxygen and supplemented by nitrogen, and water medium as the discharge medium. In this technology, the oxygen in the medium is used for exothermic oxidation, and the vaporization and explosion of the water generates a chip removal force for highly efficient erosion. The present work uses single-factor tests to compare the characteristics of processing the difficult-to-machine material titanium-alloy special-shaped cavities using either DMA-DAP or EDM. The current, pulse width, pulse interval, and dielectric pressure are selected as the single-factor processing parameters, and how they influence the material removal rate (MRR), electrode relative wear rate (ERWR) and the surface morphology of the processed square cavities is analyzed. The results show that with DMA-DAP, the MRR is more than 12 times that of EDM, the ERWR is reduced by more than 98%, and the surface morphology is relatively good. Finally, taking an aero-engine radial diffuser as the profiling object, DMA-DAP realizes a profiling sample in the form of a variable-cross-section cavity that EDM cannot process, and the efficient die-sinking processing ability of DMA-DAP is verified.

## Introduction

In order to improve the comprehensive performance, aerospace engines and nuclear industrial equipment are increasingly using integral components with new structures and new materials. However, the cavity and surface of some integral components have poor machinability, and contain deep, narrow and complex special-shaped cavities. Most of their materials are difficult to cut materials such as titanium alloy and superalloy^1–4^. Electrical discharge machining (EDM) is one of the most promising processing methods for machining difficult-to-machine materials by electric corrosion, and it plays an important role in the aerospace, die, medical, and automobile fields, among others^5–8^. However, the processing efficiency of traditional EDM is relatively low, fire must be prevented and it is easy to produce gases that could harm the environment and the operators. To tackle such problems and improve the machining efficiency, various EDM variants have been explored. Near-dry EDM is an environmentally friendly EDM method that has relatively high material removal rate (MRR), low electrode relative wear rate (EWR), and high surface quality^9,10^.

Tanimura et al.^11^ proposed a near-dry EDM technology that uses as its discharge medium water mist comprising water and air, this has the same environmentally friendly characteristics as dry EDM and also gives relatively high MRR and surface quality^12,13^. By adding oxygen into the medium of near-dry EDM, Yadav et al.^14^ found that the MRR was 203%, 110%, and 85% higher than that obtained with dry EDM using air only, dry EDM using air and oxygen, and rotary-tool near-dry EDM without oxygen, respectively. Tao et al.^15^ obtained a mirrored machined surface by using quasi-dry EDM technology. Dhakar et al.^16^ investigated how the machining parameters influenced the performance of quasi-dry EDM, and the results showed that (i) they had a significant impact on the MRR and (ii) the wear of electrode could almost be ignored. Singh et al.^17^ used the gas mist formed by argon and glycerol as the EDM medium; the MRR was improved, and a machined surface with almost no recast layer was obtained. Yadav et al.^18^ realized through-hole machining with a length of 24 mm on steel materials by using rotary-electrode near-dry EDM. Fujiki et al.^19^ established a new inter-electrode control system for near-dry EDM, which improved the MRR by nearly 30%.

The MRR of near-dry EDM is found to depend on the pulse discharge energy: the higher the energy, the higher the MRR. Also, the research object of the processing method is almost always steel, not titanium alloys, superalloys, or other difficult-to-machine materials. Kong et al.^20^ studied a difficult-to-machine titanium alloy in submersed gas-flushing EDM and found that the MRR and surface quality with an argon medium were better than those with an air medium. Meanwhile, Kong et al.^21,22^ proposed a titanium-alloy mixed-gas discharge ablation processing method based on discharge-induced ablation processing technology^23,24^ with oxygen as the main component and nitrogen or argon as auxiliary gases. The chemical energy generated by the reaction between oxygen and the titanium alloy greatly improved the rate of titanium alloy. Gas media such as nitrogen or argon with controllable oxygen concentrations were used to achieve high efficiency and stability throughout the titanium alloy processing sequence. However, this machining method is similar to dry EDM, the material removal rate, forming accuracy and surface quality need to be further improved.

To that end, this paper explores an efficient titanium-alloy die-sinking electrical discharge technology based on our previous research results, that is, die-sinking mixed-gas atomization discharge ablation process (DMA-DAP) technology—to obtain the advantages similar to near-dry EDM technology. The mixture gas and working fluid are mixed at high pressure to form an atomization medium. The atomization medium is used to improve the ablation processing state between the electrodes in order to improve the ablation formability of the titanium alloy. First, the principles of DMA-DAP are introduced, and then single-factor tests are used to study and compare the forming characteristics of DMA-DAP and traditional EDM for titanium alloys. A radial diffuser in an aero-engine is then taken as the simulation processing object to (i) realize the machining of titanium-alloy cavities with large depth ratio and variable cavity cross section and (ii) verify the efficient die-sinking processing ability of DMA-DAP.

## Methods and materials

### The principle of DMA-DAP

**Figure 1:**
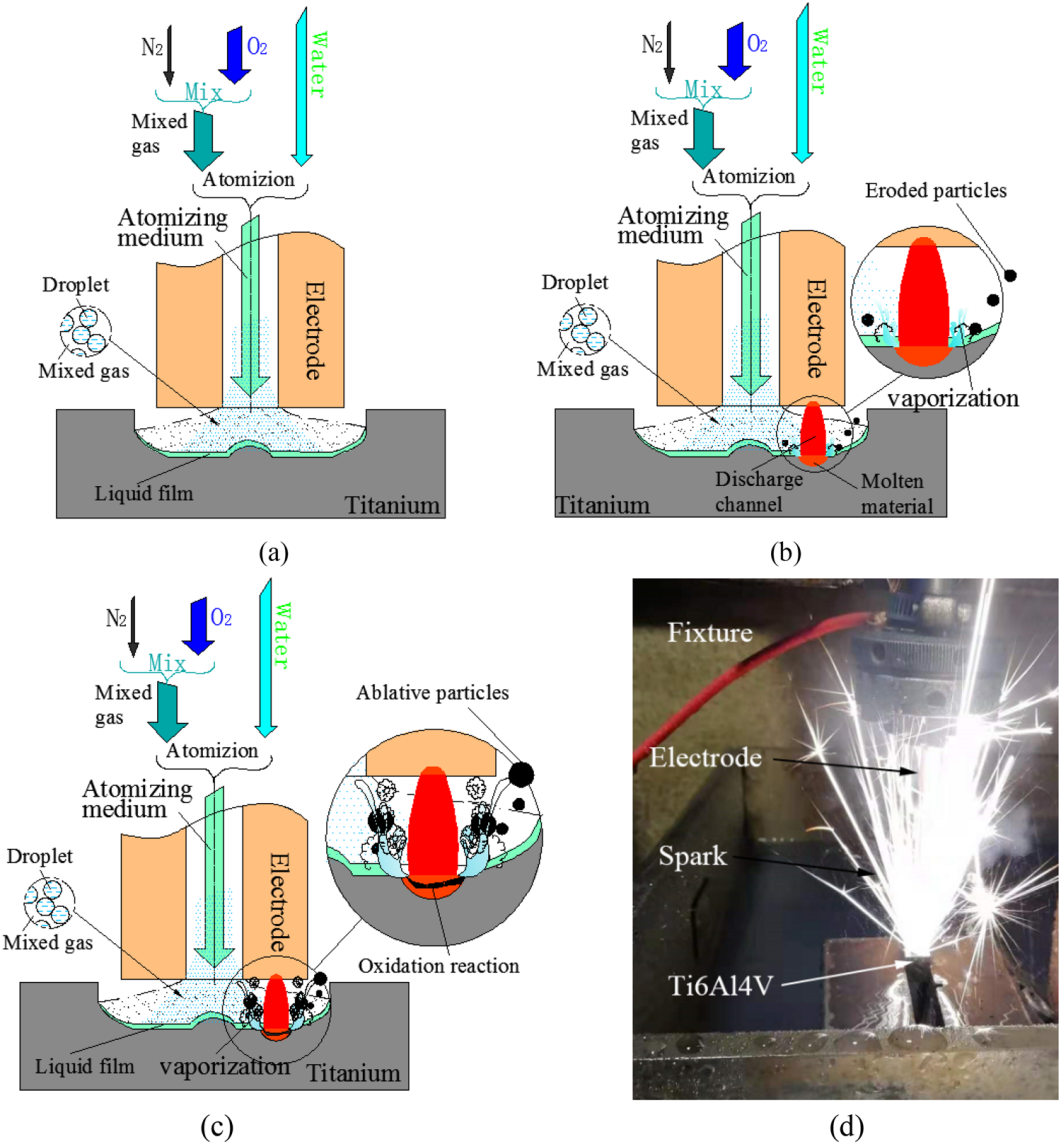
Principles of DMA-DAP: (**a**) state of inter-electrode atomizing medium; (**b**) spark discharge; (**c**) oxidation ablation and liquid vaporization; (**d**) the MA-DAP machining status.

The principles of DMA-DAP are shown in Fig. 1. In this technology, the mixed gas medium comprising oxygen and nitrogen or argon and other auxiliary gases is atomized with liquid water under high pressure to form the flow of a two-phase medium (atomization medium) with gas as the continuous phase and liquid as the discrete phase, which is led into the discharge gap through the hollow forming electrode. As shown in Fig. 1a, when water is sprayed onto the workpiece at high speed, it adheres to the workpiece under its own physical and chemical actions. As shown in Fig. 1b, when a voltage is applied between the two electrodes, the charged particles break down the discharge medium (atomization medium) to form a discharge channel, bombard the surface of the workpiece for material erosion, and form a molten pool containing high-temperature molten material at the discharge point; when the water is heated, it vaporizes. Having formed, the high-temperature molten material has a violent oxidative exothermic reaction with the oxygen in the inter-electrode mixed gas medium, thereby increasing the heat energy of the titanium alloy and realizing rapid erosion of the material. At this time, under the dual actions of the discharge channel and the oxidation exothermic high temperature, the water vaporizes rapidly and expands until it explodes, thereby indirectly increasing the discharge power of the products from the inter-electrode corrosion, as shown in Fig. 1c. The main purpose of the nitrogen or argon in the mixed gas is to reduce the oxygen concentration and control the stability of the ablation process^28,29^. Figure 1d shows the actual machining status of the MA-DAP. During machining, the titanium alloy combustion spark fills the entire discharge area and the ablation products are discharged in the form of an eruption.

**Figure 2:**
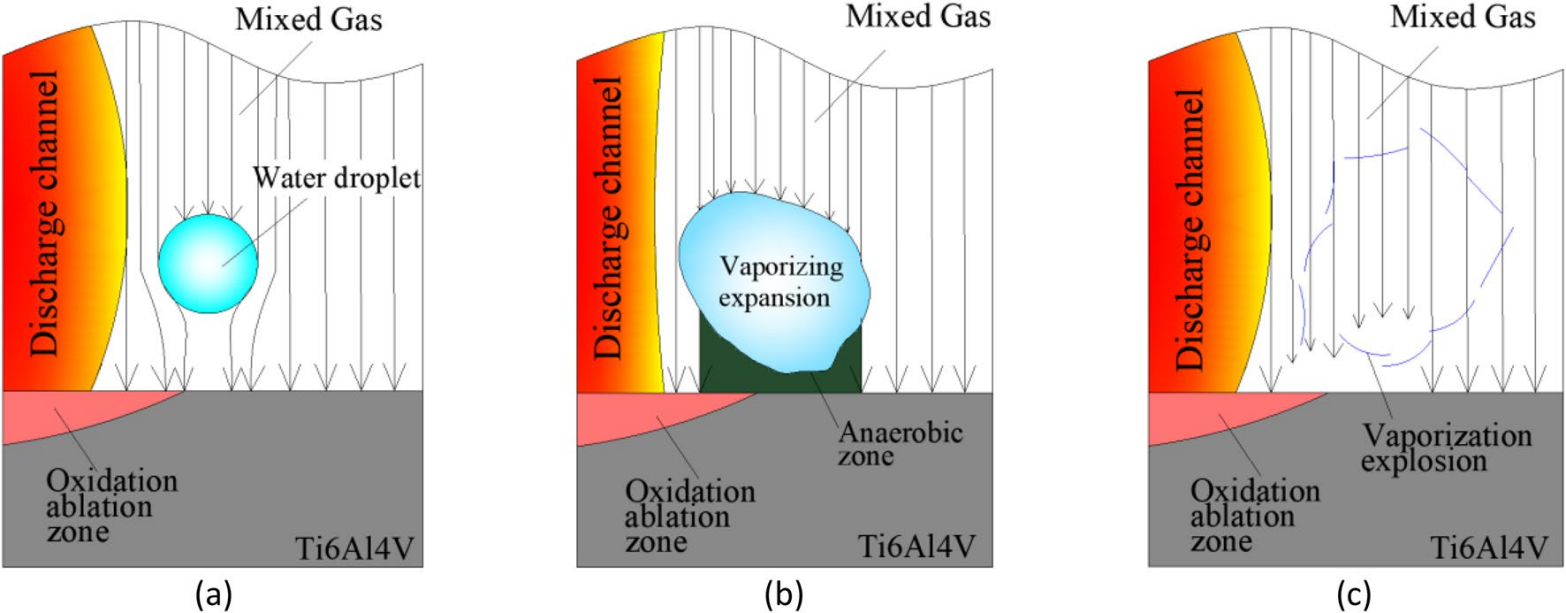
Schematics of droplet vaporization: (**a**) water droplet; (**b**) vaporization; (**c**) explosion.

According to thermodynamics, water medium will change from liquid phase to gas phase under the high temperature between the discharge electrodes, resulting in volume change. Water is vaporized completely at 100 °C, and the volume ${\mathrm{V}}_{v}$ of water between the electrodes after complete vaporization is

1 $$V_{v} = \frac{{1000 \times V_{L} \times K}}{M} = \frac{{1000 \times V_{L} \times 22.4}}{18} = 1244.44V_{L}$$

where K (L/mol) is the molar mass of gas, M (g/mol) is the molar mass of water, and *V*_*L*_ (L) is the volume of water in the discharge gap. For a narrow discharge gap, the water vapor squeezes the surrounding space and produces a greater driving force on the eroded particles, thus discharging the corrosion products rapidly from the inter-electrode discharge gap. The presence of water in the atomizing medium not only increases the chip removal force but also has a favorable impact on the discharge channel between the electrodes and the oxygen supply environment. The process of vaporizing and expanding to explosion when the water droplets in the electrode are heated is shown in Fig. 2.

From the beginning of the DMA-DAP to its end, the water droplets are always in an environment that is much hotter than the temperature required to vaporize them, as shown in Fig. 2a. From thermodynamics, we know that the water droplets expand sharply after being heated, as shown in Fig. 2b. At this time, the mixed gas (including oxygen) around the water droplets is replaced by water vapor, thereby hindering contact between the oxygen and the high-temperature molten material. When the water droplets vaporize and expand to a certain limit, an explosion occurs. As shown in Fig. 2c, the space occupied by their expansion is replaced by the mixed gas medium. Oxygen comes into contact with the molten material on the surface of the workpiece under high pressure, whereupon violent oxidation occurs. When water droplets are in a high-temperature environment, their surface tension is extremely low and their lifespan is extremely short. The intermittent oxygen supply environment induced by the instantaneous vaporization and explosion of water droplets is conducive to disturbing the discharge channel and improving the inter-electrode discharge state. Moreover, the sharp vaporization explosion is conducive to squeezing and expelling the erosion products in the molten pool, exposing the high-temperature molten material at the bottom of the molten pool to continue to participate in the oxidation, improving the efficiency of inter-electrode oxidation, and thereby increasing the MRR.

**Figure 3:**
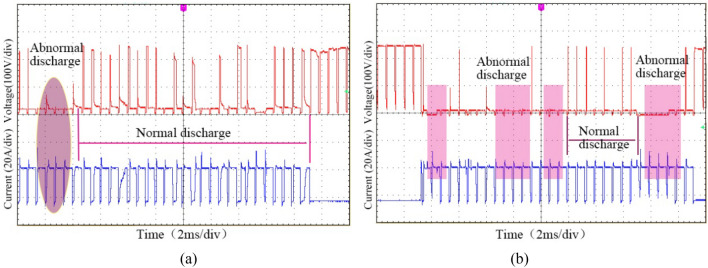
Discharge waveforms: (**a**) DMA-DAP (O_2_:N_2_ = 5:1); (**b**) electrical discharge machining (EDM).

The sharp vaporization of the water enhances the removal force acting on the erosion particles between the electrodes, which is effective for reducing the probability of corrosion products accumulating, improving the inter-electrode discharge environment, and avoiding the generation of abnormal pulse discharges such as short circuits and arcs, thereby increasing the number of normal discharge pulses. Figure 3 shows the discharge waveforms under the two processing methods. As can be seen, the effective discharge waveforms of DMA-DAP are more numerous than those of EDM, indicating that the effective discharge probability of DMA-DAP is higher, which is conducive to improving the ablation efficiency substantially.

### Experimental system and workpiece material

**Figure 4:**
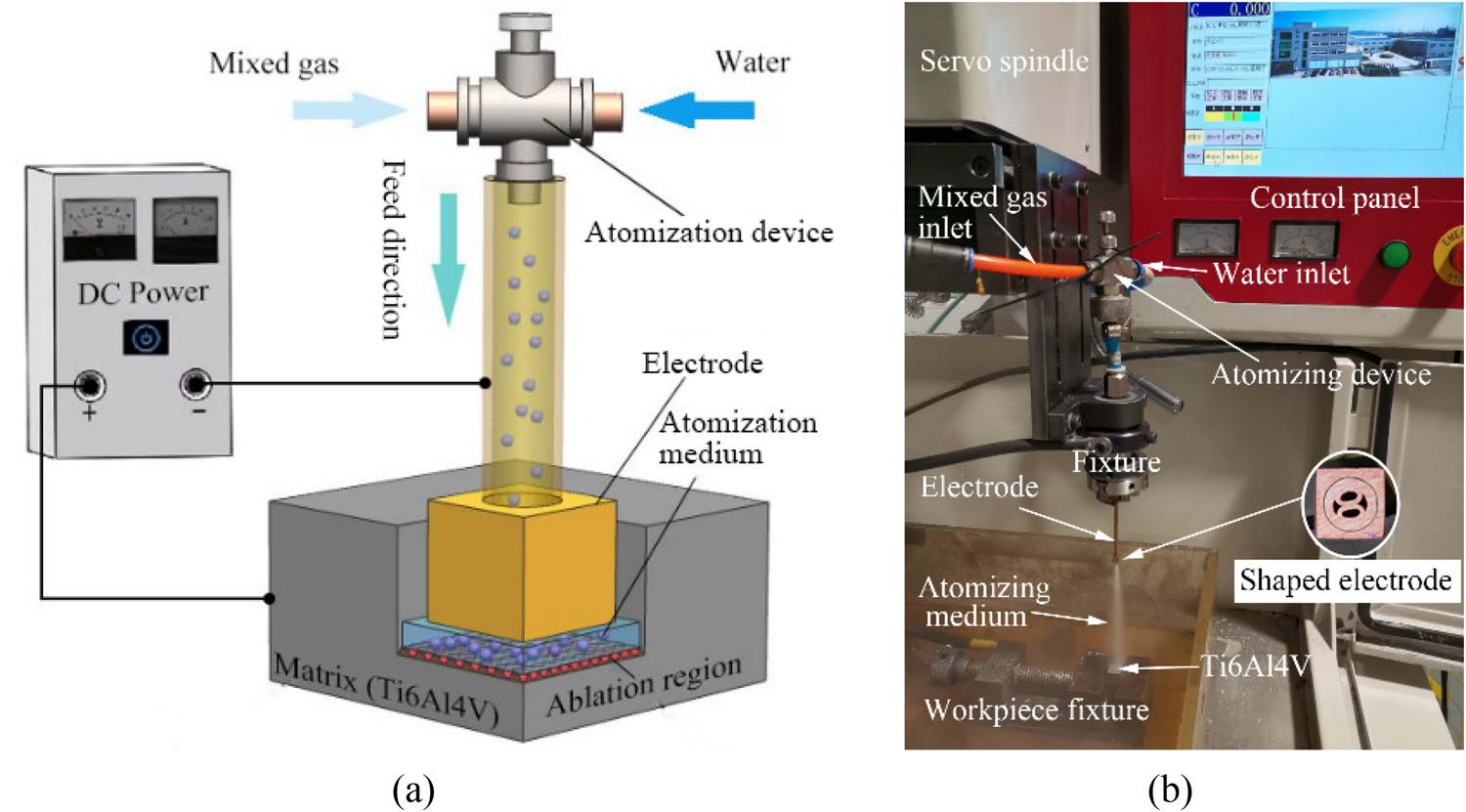
(**a**) Schematic and (**b**) actual experimental setup of die-sinking mixed-gas atomization discharge ablation process (DMA-DAP).

In Fig. 4, the experimental system is shown schematically and as the physical device. The experiment adopts the EDM machine tool (model: NIVA NH250) produced by Beijing Ninghua Co., Ltd. by adding some attachments as the requirement of DMA-DAP process. When the system is operating, the mixed gas with a certain oxygen concentration and the high-pressure water flow mix in the atomization device to form the atomization medium. The latter is then sprayed into the processing area through the hollow electrode to participate in the discharge and ablation between the electrodes. The atomizing device and electrode are fixed to the spindle of the machine tool and perform servo feed motion.

In order to study the processing ability of DMA-DAP special-shaped cavity, a hollow copper electrode with a square end (5 mm × 5 mm) was used as the tool electrode, and the cavity depth of the titanium-alloy sample was set 5 mm. Using the processing parameters given in Table 1, the single-factor experimental method was used to compare the titanium-alloy cavity-forming characteristics of DMA-DAP and traditional EDM, and the MRR and electrode relative wear rate (ERWR) were used as the research objects. Scanning electron microscopy (SEM, Hitachi S-3400N) was used to scan the surface of the processed workpiece and analyze its element energy spectrum.

**Table 1:** Experimental conditions.

Method	DMA-DAP	EDM
Current *I* (A)	12, 15, 18	12, 15, 18
Pulse width *T*_on_ (µs)	100, 220, 320, 480, 600	100, 220, 320, 480, 600
Pulse interval *T*_off_ (µs)	60,100, 140,180, 220	60,100, 140,180, 220
Oxygen concentration *η* (O_2_:N_2_ = 6:1, pressure ratio)	η = 85.7%	×
Medium pressure *P* (MPa)	0.1, 0.2, 0.3, 0.4 (mixed-gas)	0.1, 0.2, 0.3,0.4 (EDM oil)
Atomization amount *L* (mL/min)	40	×
Polarity	Workpiece (+)	Tool electrode (−)

Titanium alloys are used widely in the aerospace, biomedical, and weapon fields, among others, because of their high specific strength, corrosion resistance, excellent high-temperature mechanical properties, and good biocompatibility. However, titanium alloys have low thermal conductivity, small specific heat, and low elastic modulus, making them typical difficult-to-machine materials^25–27^. It is difficult to process titanium-alloy cavity parts by traditional machining methods, and in particular it is almost impossible to achieve deep narrow cavities. Therefore, the workpiece material selected in this study was Ti6Al4V. The chemical composition of the titanium alloy is given in Table 2, and the salient characteristics of the workpiece are given in Table 3.

**Table 2:** Chemical composition of Ti6Al4V alloy.

Element	Ti	Al	V	Fe	O	C	N	H
wt%	89.464	6.08	4.02	0.22	0.18	0.02	0.01	0.0053

**Table 3:** Properties of Ti6Al4V.

Property	Unit	Value
Density	g/cm^3^	4.5
Elastic modulus	GPa	110
Melting point	°C	1660
Hardness	HRC	30
Thermal conductivity	W/mK	7.955

## Results and discussion

### Influence of discharge energy on MRR

**Figure 5:**
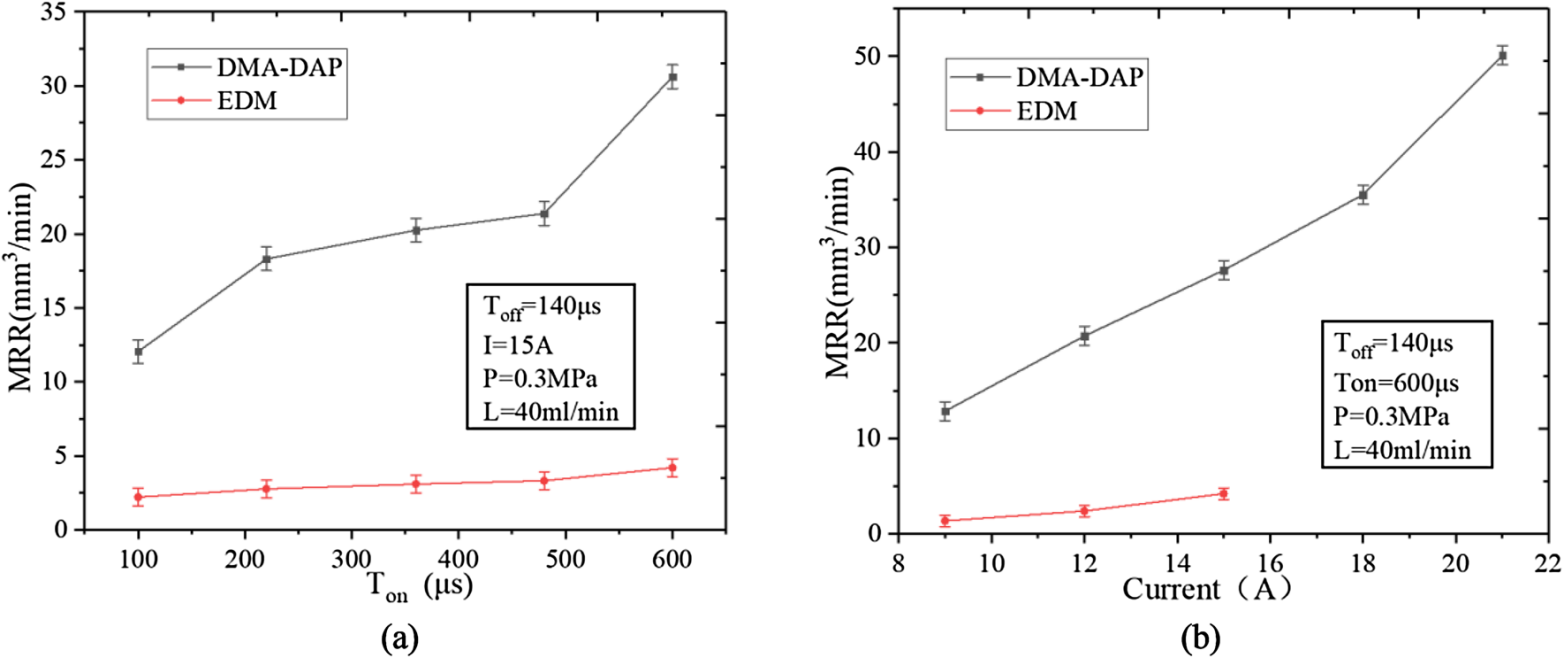
Effects of (**a**) *T*_on_ and (**b**) current on material removal rate (MRR).

For a given processing depth, the MRR of the DMA-DAP and EDM of titanium alloy increases with increasing discharge current and pulse width. As shown in Fig. 5, regardless of the discharge parameters, the MRR of the DMA-DAP of titanium alloy is more than five times that of EDM, which shows that DMA-DAP has greater advantages. In this experiment, to avoid the risk of ignition during machining, we did not operate EDM with high current.

For EDM, the current and the pulse width are the main sources of material removal energy, and increasing both increases the energy acting on the workpiece material per unit time, thereby increasing the MRR. For DMA-DAP, increasing the discharge energy increases the volume of high-temperature molten material involved in the oxidation and ablation reaction between the electrodes, thereby increasing the chemical heat energy and greatly improving the heat removal energy of the titanium alloy. Furthermore, the water in the atomization medium has a lower vaporization point than that of Oil-based working fluid, thereby accelerating the cooling and solidification of the inter-electrode corrosion products and quickly discharging the inter-electrode discharge gap under the action of the water vaporization explosion, which is conducive to improving both the discharge state between the electrodes and the MRR.

### Effects of pulse interval on MRR and ERWR

**Figure 6:**
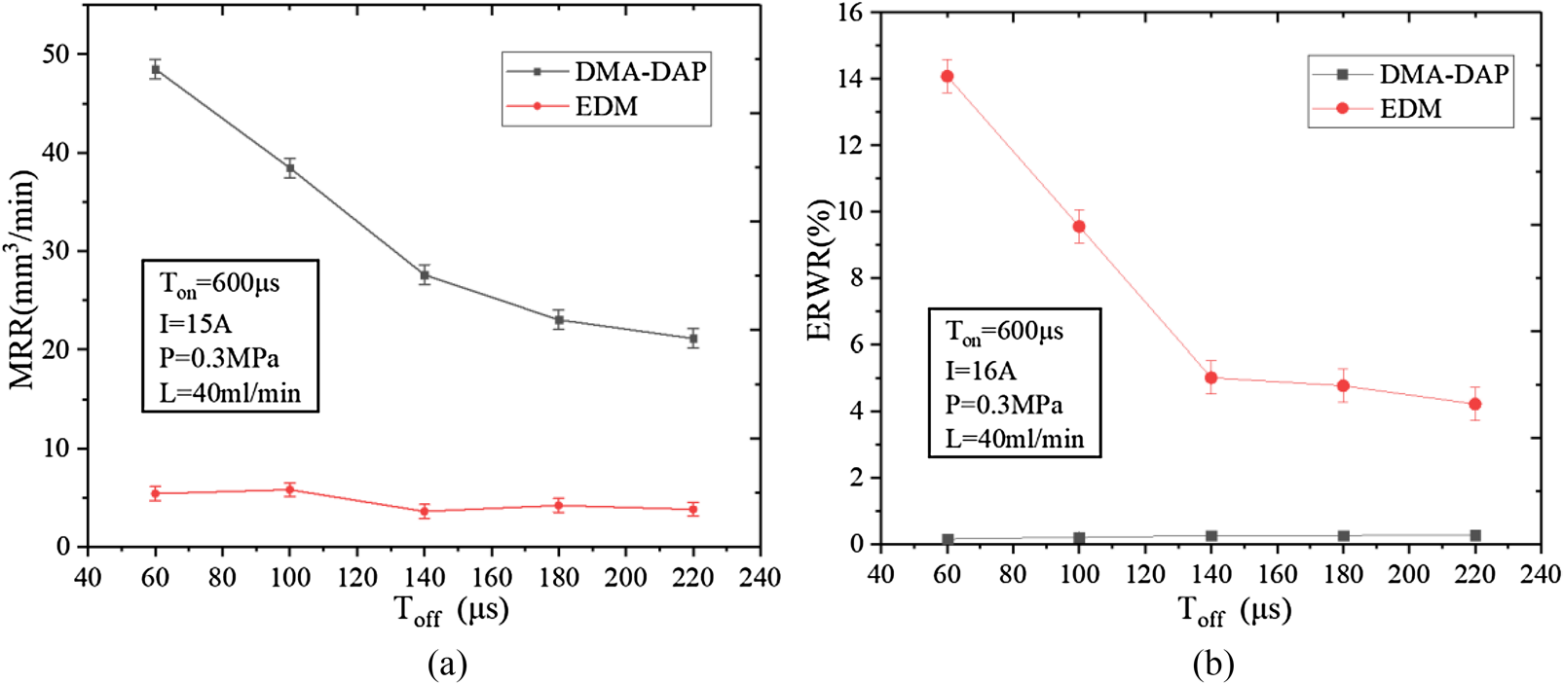
Effects of *T*_off_ on (**a**) MRR and (**b**) electrode relative wear rate (ERWR).

Figure 6 shows how the pulse interval affects the MRR and ERWR. The pulse interval affects the two processing methods very differently. When using a small pulse interval (60 μs), the MRR of DMA-DAP is nearly 10 times that of EDM, while the ERWR is reduced by more than 98%; when using a large pulse interval (220 μs), the MRR of DMA-DAP is more than five times that of EDM, while the ERWR is reduced by 93%. The analysis shows that the smaller the pulse interval, the less favorable the elimination of ionization between the electrodes. The corrosion products after discharge have no time to diffuse and discharge in a short period of time, and the heat in the discharge channel cannot be transmitted in time, thereby decreasing the dielectric property of the working medium and leading to frequent abnormal discharges between the electrodes, thereby increasing the electrode wear.

For DMA-DAP, the MRR decreases with increasing pulse interval, whereas the ERWR changes by less than 0.5%. The violent oxidation and ablation reaction and the explosive force generated by the vaporization of the water between the electrodes interfere strongly with the discharge channels, which helps the deionization of the pulses between the electrodes. Reducing the pulse interval increases the discharge energy density between the electrodes, which indirectly increases the volume of high-temperature molten material involved in the oxidation and ablation reaction, and increases the chemical energy in the discharge gap, thereby increasing the MRR. In DMA-DAP, the high-speed airflow and the water vaporization that is endothermic on the electrode accelerate the heat diffusion on the electrode. The results show that the discharge gap between the electrodes is small, and the sharp oxidation and ablation reaction causes the corrosion products to adhere to the electrode surface. At the same time, the water expands rapidly and explodes, causing the corrosion products to splash onto the electrode surface and form a protective film, thereby decreasing the electrode loss of DMA-DAP.

### Effects of medium pressure on MRR

**Figure 7:**
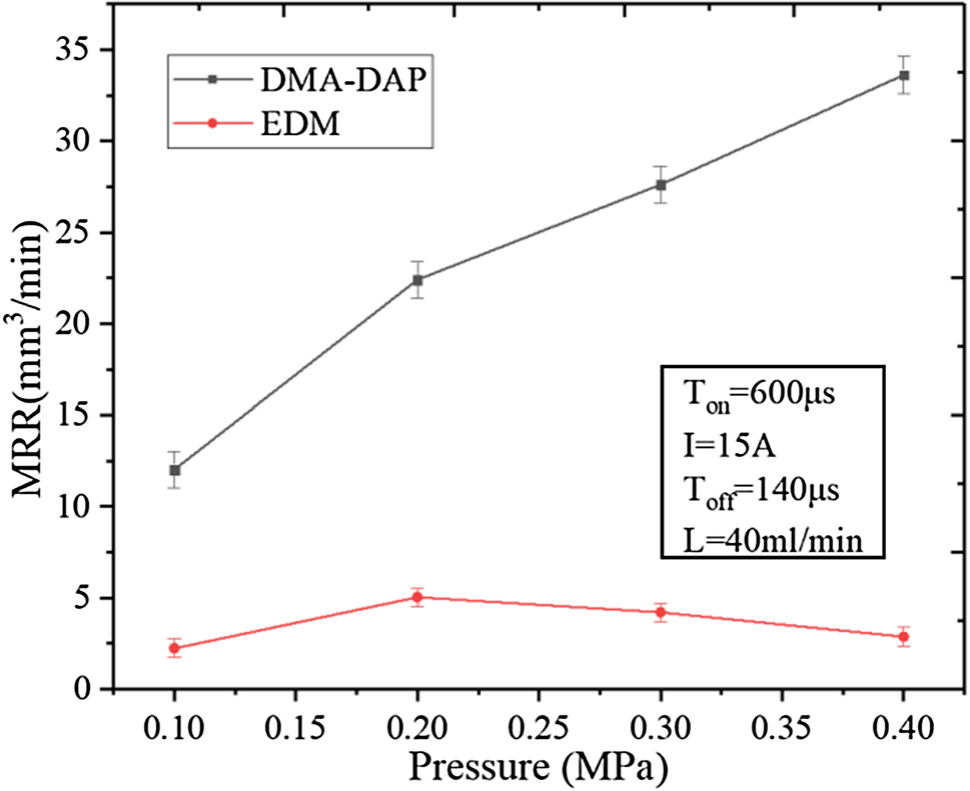
Effect of medium pressure on MRR.

Figure 7 shows how the medium pressure affects the MRR of the two machining methods. The MRR of DMA-DAP is always higher than that of EDM, and the difference increases with increasing medium pressure. When the medium pressure is 0.4 MPa, the MRR of DMA-DAP is nearly 12 times that of EDM. The MRR of DMA-DAP increases with increasing gas pressure. The research results of Kong et al.^27,28^ show that increasing the gas pressure helps to (i) increase the diffusion speed of oxygen to the matrix, (ii) increase the amount of oxygen participating in the oxidation and ablation reaction in unit time between the electrodes, (iii) intensify the release of chemical energy between the electrodes, and (iv) increase the MRR. Also, increasing the gas pressure helps to increase the chip removal force of the inter-electrode ablation products, and the latter can be discharged from the discharge gap in time to improve the discharge state of the atomization ablation process, thereby increasing the probability of discharge ablation.

### Comparison of surface topography

**Figure 8:**
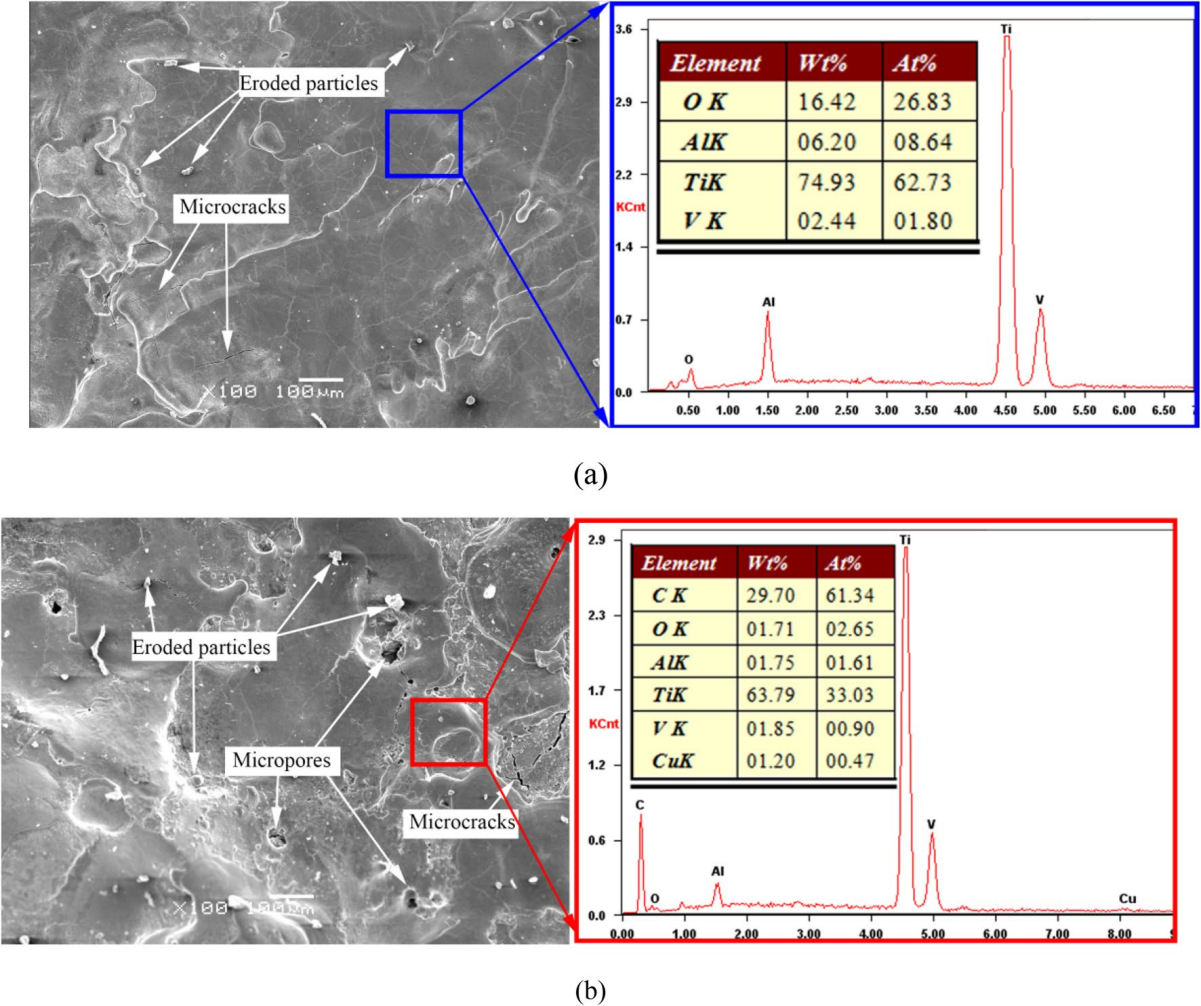
Scanning electron microscopy images and energy-dispersive X-ray spectroscopy spectra surfaces of machined by (**a**) DMA-DAP and (**b**) EDM.

The surface quality of the workpiece is very important for its use. Figure 8 shows the surface morphology and corresponding surface energy spectrum of samples processed by the two methods. The surface processed by DMA-DAP is relatively smooth and flat and contains a small amount of cooling and solidification accumulation of melt-eroded products. In contrast, the surface processed by EDM contains more microcracks and micropores. Furthermore, unlike the surface of the EDM sample, that of the DMA-DAP sample contains no carbon and the oxygen content is relatively high. This shows that the main product formed on the surface of the DMA-DAP sample is the oxide of the titanium alloy, whereas the main product formed on the surface of the EDM sample is the carbide of the titanium alloy. The melting point or specific heat of titanium-alloy oxide is lower than that of titanium-alloy carbide, and the energy required is relatively low in the actual etching process, which is one of the reasons for the high MRR in DMA-DAP. The surface of the EDM sample also contains a certain amount of copper, which indicates that the copper electrode material melted and sputtered onto the sample surface because of the influence of abnormal discharge between the electrodes during machining, which also explains the relatively high ERWR of the EDM electrode.

### Comparison of surface roughness

In order to evaluate the surface roughness of the two machining processes, this paper adopts the surface roughness Sa, which is more suitable for evaluating the surface roughness in the microscopic field. Sa is the roughness evaluation parameter based on the regional morphology, and its mathematical expression is:

2 $${s}_{a}=\frac{1}{NM}\sum_{i=1}^{N}\sum_{j=1}^{M}|{Z}_{ij}|$$

where is the distance from the point on the contour of the Z object surface area to the reference plane, and M and N are the number sampling points in two directions perpendicular to each other in the evaluation area. It represents the arithmetic mean deviation of regional morphology and is used to characterize the roughness of three-dimensional morphology of object surface.

**Figure 9:**
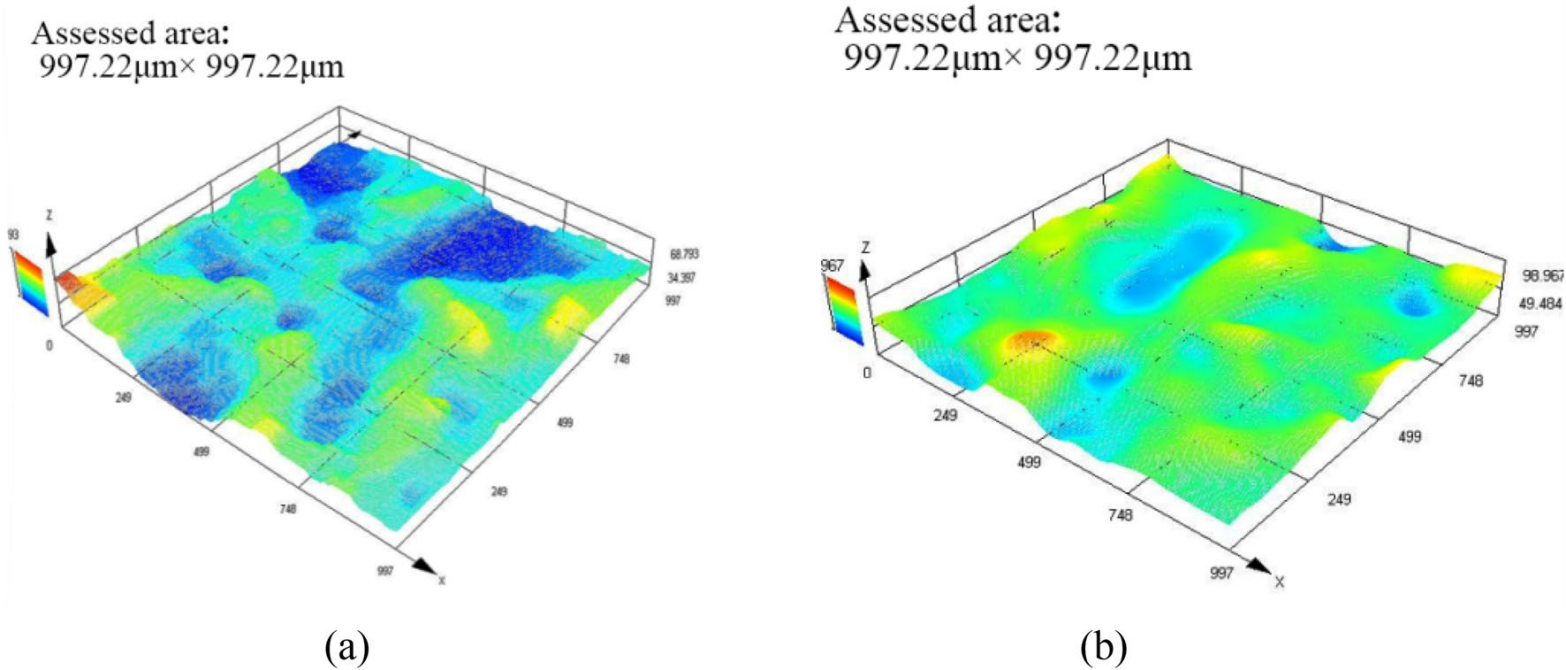
The surface roughness (Sa) of (**a**) DMA-DAP and (**b**) EDM.

Figure 9 shows the surface roughness Sa of the two processing methods measured by the professional measuring instrument OLYMPUS. The Sa of DMA-DAP is 4.52 and the Sa of EDM is 7.91 μm. The surface roughness value (Sa) of the former is reduced by nearly 43% compared with the latter. It can be seen that although the material removal rate of DMA-DAP has increased, its surface quality has not been reduced, but has improved compared with EDM. The analysis suggests that the High dielectric fluid viscosity of EDM's spark oil medium has a greater compression effect on the discharge channel, resulting in a deeper discharge pit on the surface of the workpiece, and its High dielectric fluid thermal conductivity aids molten metal material rapidly solidifies around the discharge crater, resulting in high surface roughness. However, the dielectric fluid viscosity and dielectric fluid thermal of the gas–liquid two-phase medium of DMA-DAP are relatively low, and the resulting discharge carter is relatively flat. In addition, the rapid vaporization and explosion of the liquid-phase granular medium generates a large thrust on the inter-electrode gap molten metal materials, which can reduce the accumulation of molten metal on the workpiece surface, and finally form a high-quality machined surface.

### Machining comparison of variable-cross-section cavity sample

**Figure 10:**
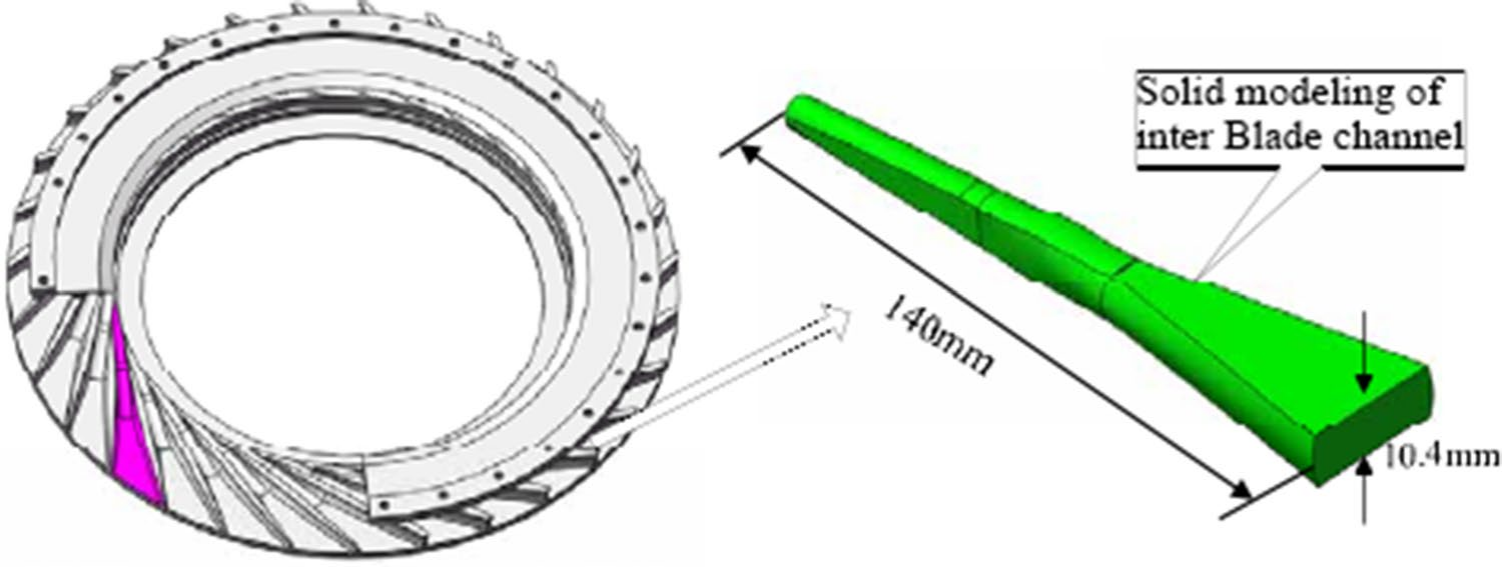
Model of radial diffuser^28^.

Figure 10 shows a radial diffuser that is used commonly in aero-engines. Most of the materials therein are difficult-to-machine ones such as titanium alloy and high-temperature alloys. The flow channel structure of the parts is characterized by being deep and narrow and having large curvature change, variable and complex cross section, and thin walls, among other aspects. Using traditional machining tools, the wear is large and the accessibility is poor, thereby making processing difficult. Currently, computer-numerical-control EDM is the main method used to machine such parts, but EDM has problems such as low efficiency and high electrode loss. Therefore, herein we take this type of variable-cross-section specially shaped cavity part as the object of profiling processing to assess the forming ability and processing efficiency of DMA-DAP.

**Figure 11:**
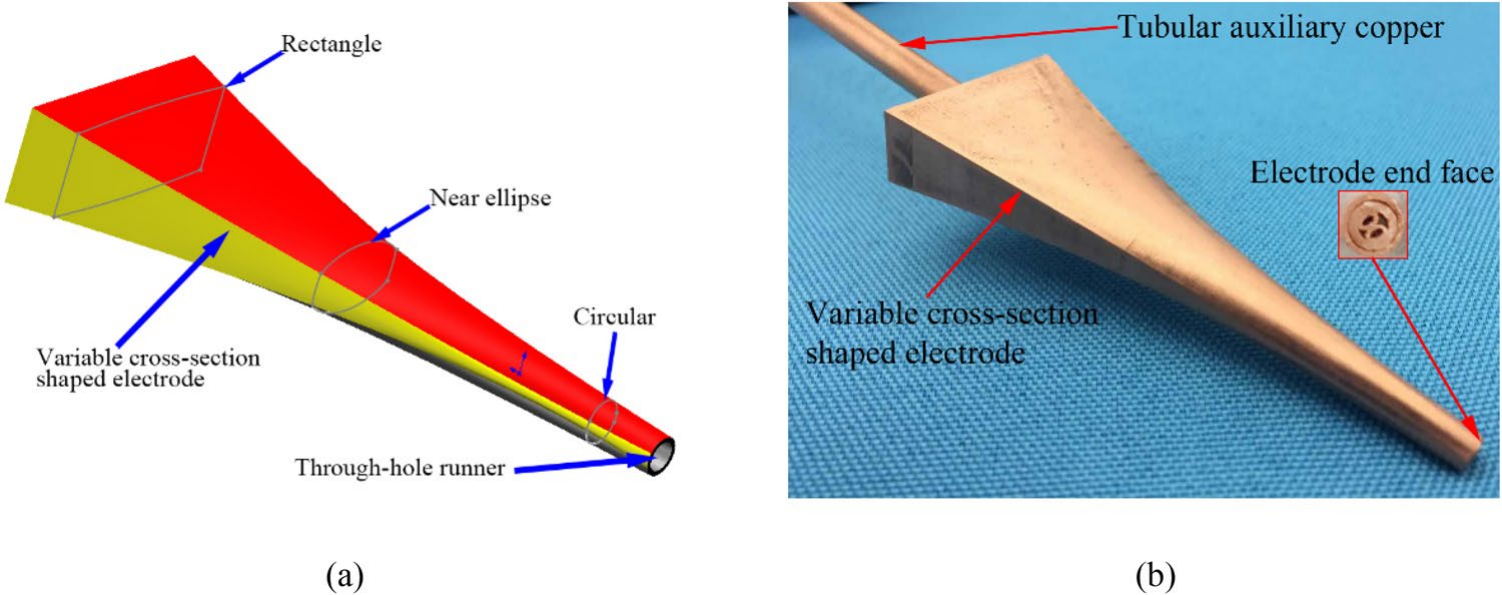
(**a**) Model and (**b**) actual sample of profiling electrode.

As shown in Fig. 11a, the variable-cross-section specially shaped electrode for the profiling processing of this type of part comprises three cross-sectional shapes—rectangular, nearly elliptical, and circular—with a through-hole channel at the center to allow the dielectric to enter the inter-electrode discharge gap. The actual electrode is shown in Fig. 11b.

**Figure 12:**
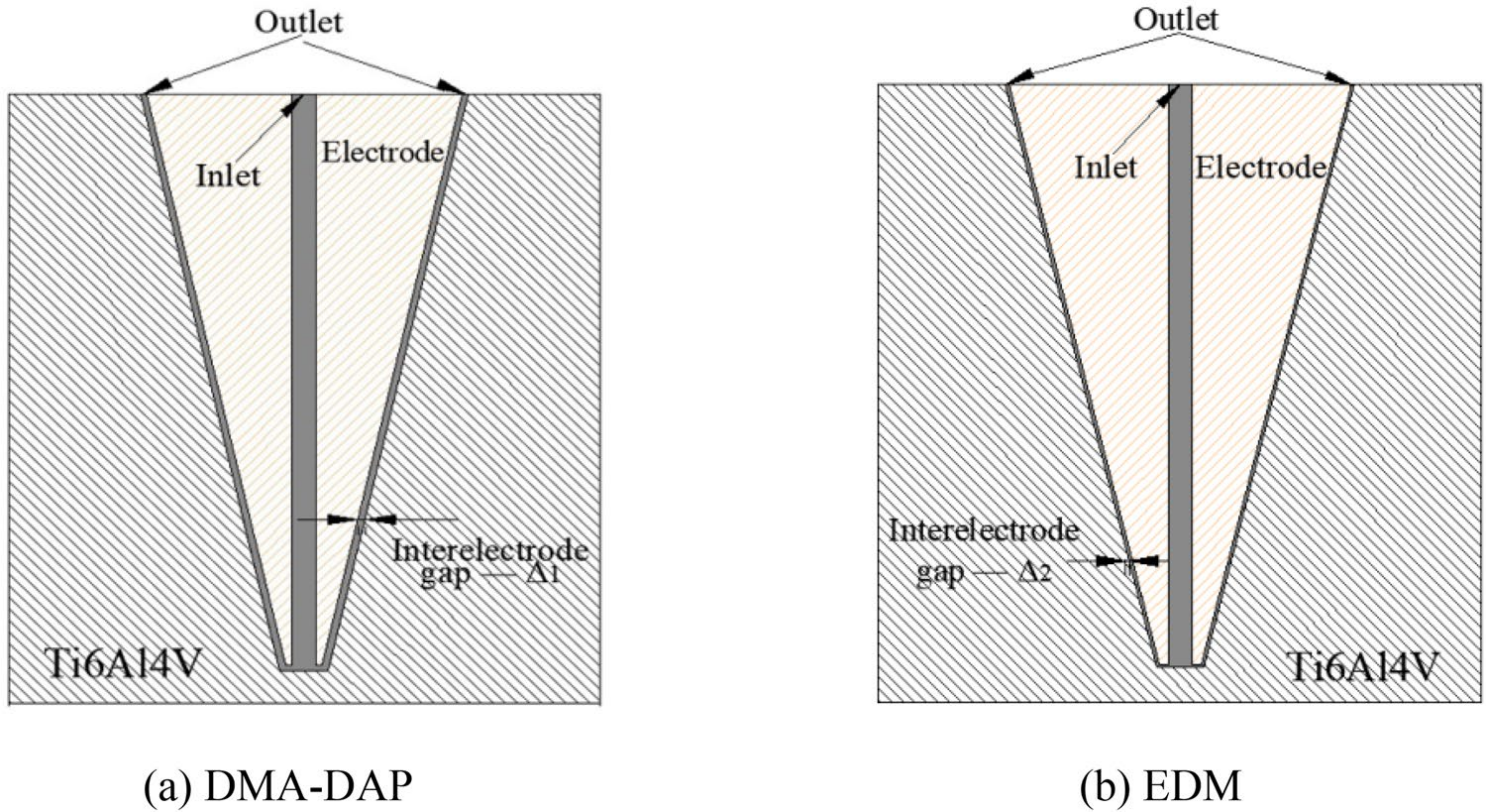
The simulation model.

**Figure 13:**
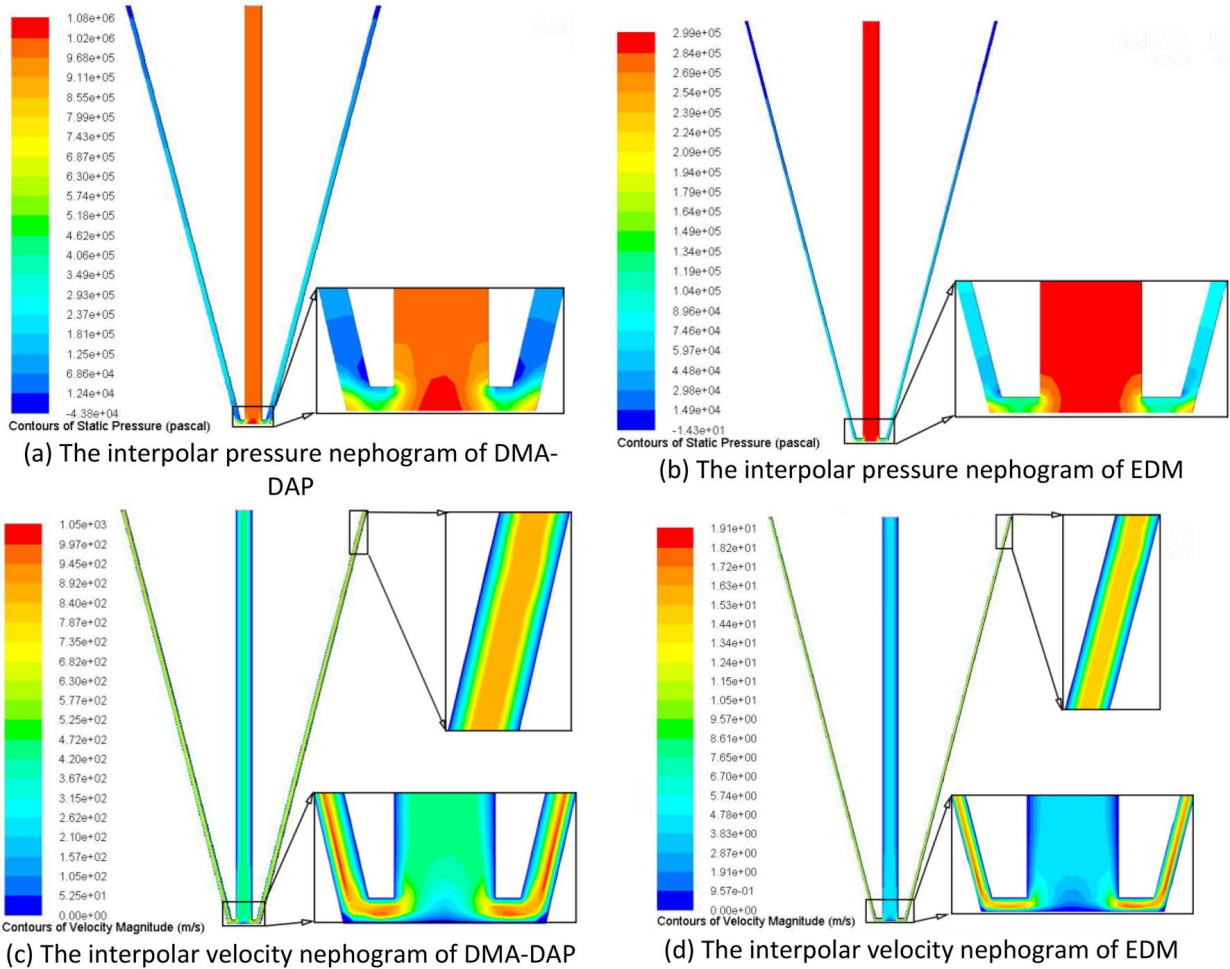
The simulation results of flow field.

In order to have a clear understanding on flushing condition in the gap, simulation of fluid flow is implemented. The reason is to find out fluid distribution between tool bottom face and workpiece in DMA-DAP and traditional EDM. Simulation is conducted with the commercial software ANSYS. According to the research of Krishnakant et al.^29^, using gas–liquid two-phase medium as discharge medium can induce the increase of inter-electrode discharge gap. Therefore, the discharge gap ∆_1_ of DMA-DAP constructed in this paper is greater than ∆_2_ of EDM. The specific two-dimensional simulation model is shown in Fig. 12. In our previous research literature^30^, the rapid vaporization and expansion of the liquid medium will cause the inter-electrode pressure to be 2.7 times the gas supply pressure, that is, the inter-electrode pressure reaches 3.7 times the gas supply pressure. Therefore, during the simulation process, the pressure inlet of DMA-DAP is set to 1.11 MPa, the fluid medium is gas, the inlet pressure of traditional EDM is set to 0.3 MPa, the fluid medium is spark oil, and the outlet pressure was environmental pressure. The k − ε turbulent model was selected as the calculation model based on the Reynolds number (Re). Figure 13 shows the comparison results of pressure and velocity nephogram of the two processing methods.

Compared with traditional EDM, due to the explosive force generated by the vaporization of the liquid particles between the electrodes, the discharge gap of DMA-DAP can obtain a higher chip removal pressure. Figure 13a and b show that the pressure difference between the two processing methods is large, which also means that as the processing depth of the processed sample increases, the erosion particles produced by DMA-DAP processing are more likely to be discharged out of the discharge gap to obtain a better inter-electrode discharge state. During EDM processing, the discharge gap between the electrodes is small. As the processing depth increases, the pressure between the electrodes is attenuated, which affects the discharge of eroded particles and is not conducive to the stability of electrical discharge machining. Figure 13c and d show the comparison of the velocity nephogram of the corresponding two processing method. It can be seen that the fluid velocity in DMA-DAP is much higher than that in EDM, whether in the bottom gap or the side wall outlet gap. The increase of fluid velocity can effectively bring the etched particles out of the discharge gap, especially the cavity sidewall gap, which can reduce the adhesion of etched particles and avoid the occurrence of secondary discharge field between electrodes, so as to obtain a stable discharge machining process.

**Figure 14:**
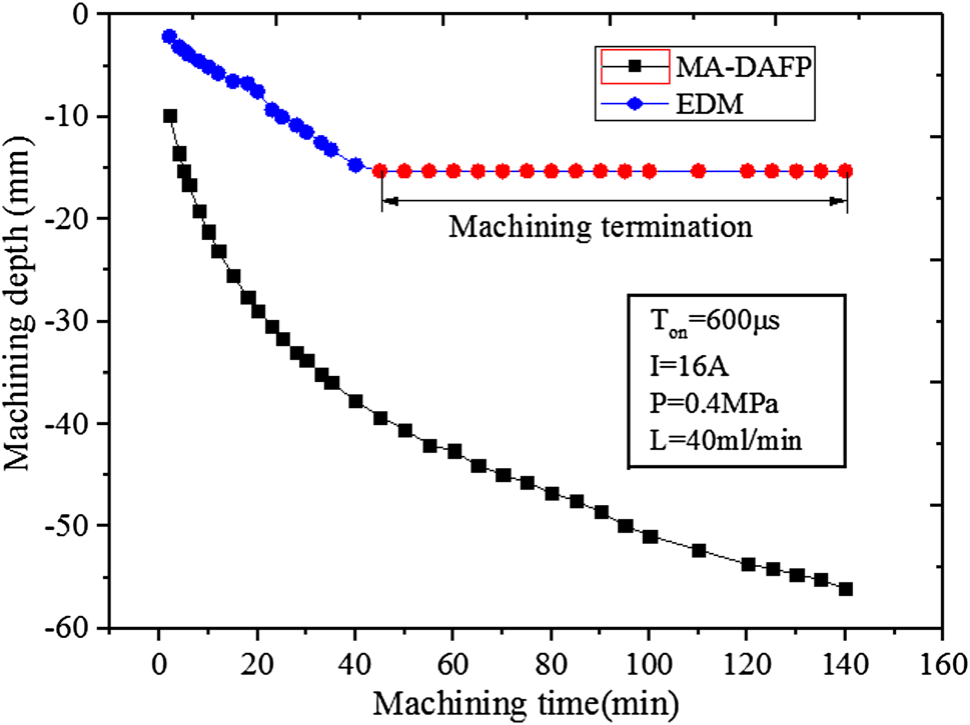
The machining process of variable-cross-section cavity.

**Figure 15:**
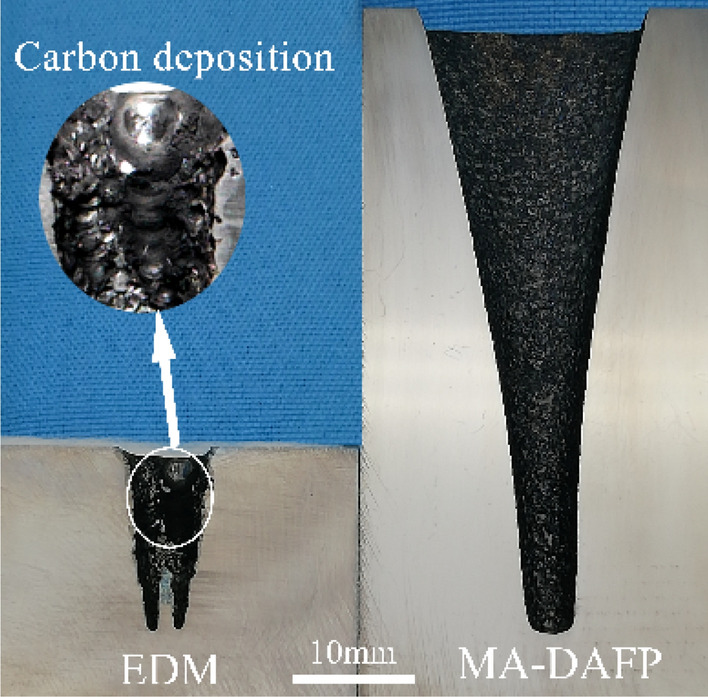
Variable-cross-section cavity samples.

According to the above single-factor test results, the MRR of DMA-DAP increases with increasing discharge energy (current, pulse width) and medium pressure: the smaller the pulse interval, the higher the efficiency. Therefore, the selected processing parameters and actual processing process were as shown in Fig. 14, and the actual variable-cross-section cavities processed by the two methods are shown in Fig. 15.

Figure 14 shows that the machining speed of DMA-DAP is much higher than that of EDM. With EDM, it took 45 min for the machining depth to reach − 15 mm; during the machining, tool lifting and arc pulling occurred frequently, and the machining had to be terminated. However, with DMA-DAP, it took only 10 min for the machining depth to reach 15 mm, and no tool lifting occurred during the atomization ablation for that 15 mm. With DMA-DAP, the final machining depth exceeded 55 mm, much more than that of traditional EDM, which shows that DMA-DAP has higher material removal and forming ability for titanium alloy. It also shows that the proposed processing technology can be used as a highly efficient machining procedure for titanium-alloy cavity parts.

As the processing depth of EDM increases, the area of formed electrode participating in the discharge machining increases, and the electrode side wall also participates in the inter-electrode discharge etching process, which makes it more difficult to discharge the corrosion products at the bottom of the electrode. When the molten corrosion products cannot be discharged in time, carbon black nodules (as shown in Fig. 15) are likely to form at the exit of the workpiece and adhere to its surface, causing the entire discharge circuit to short-circuit, arcing, and other abnormal discharge phenomena, thereby making it impossible for EDM to continue normal processing. With DMA-DAP, the vaporization expansion and chip removal function of the atomizing medium mean that the whole process is relatively stable, and there are no abnormal processing phenomena such as short-circuits and arc drawing. However, it can also be seen that as the processing time increases (i.e., the processing depth increases), the processing speed gradually decreases. The analysis shows that with increasing machining depth, the area of variable-cross-section electrode participating in the discharge increases. Under a given discharge-induced energy, increasing discharge area means that the ablation energy density of unit discharge between the electrodes decreases, the ablation state of the inter-electrode discharge deteriorates, and the stability of the ablation processing decreases, thereby decreasing the machining efficiency. Furthermore, although the atomizing medium has a strong vaporization chip removal effect, with increasing discharge area of the electrode side, the chip removal channel at the bottom of the electrode is extended, thereby increasing the discharge resistance of the etched products and in turn decreasing the efficiency of discharge ablation.

**Figure 16:**
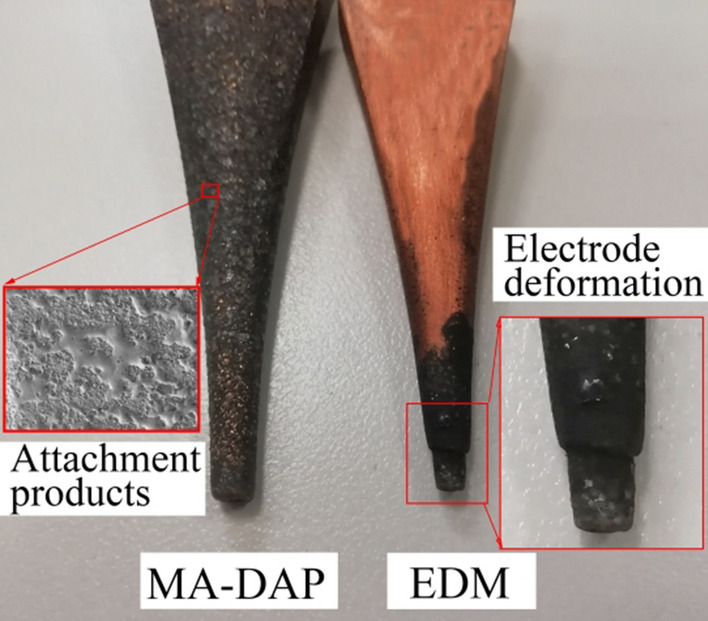
Profile electrodes after processing variable-cross-section cavities.

Figure 16 shows the formed electrodes after machining. As can be seen, the EDM electrode was seriously deformed, this being because of the occurrence of arcing or short-circuits caused by the failure to remove products from the inter-electrode gap in time, which indirectly increases electrode wear. However, with DMA-DAP, the products of inter-electrode erosion adhere to the surface of the profiling electrode and form a protective layer thereon, which reduces electrode wear and ensures the shape accuracy of variable-cross-section cavity machining.

## Conclusions

In this study, an efficient electrical machining technology known as DMA-DAP was proposed. The conclusions are as follows.

1. In DMA-DAP, the water in the atomization medium is subjected to the double high-temperature action of the discharge channel and the oxidation and ablation reaction, and a rapid vaporization explosion occurs, which greatly improves the discharge state between the electrodes. This is effective in reducing the occurrence of abnormal discharge such as short circuit and increasing the probability of normal electrical discharge.
2. The MRR of DMA-DAP increases with increasing current, pulse width, and mixed-gas pressure and with decreasing pulse interval, allowing values of the MRR as high as 12 times that of EDM. The ERWR changes little with increasing pulse interval, and compared with EDM, it can be reduced by more than 98%. Therefore, high energy, high medium pressure, and small pulse interval should be used for high-efficiency machining of titanium-alloy cavities.
3. Compared with EDM, the surface morphology with DMA-DAP is smoother. At the same time, the surface roughness (Sa) value after DMA-DAP processing is reduced by 43% compared with EDM. In addition, titanium-alloy oxides with lower melting point form on the surface during the DMA-DAP machining process, whereas the EDM surface is a titanium-alloy carbide with relatively high melting point and that requires more energy, which is one of the factors leading to the relatively high MRR of DMA-DAP.
4. DMA-DAP realizes the high-efficiency machining of variable-cross-section diffuser cavity sample parts similar to aviation components, which cannot be realized by EDM. The experimental results show that DMA-DAP has strong forming ability and is suitable for high-efficiency machining of titanium-alloy special-shaped cavity parts.
